# (4a*S*,5*R*,7*R*,8*S*,8a*R*)-8-(1,3-Dioxolan-2-yl)-7,8-dimethyl-5-(1-methyl­ethen­yl)perhydro­naphthalen-2-one

**DOI:** 10.1107/S1600536807067335

**Published:** 2007-12-21

**Authors:** Maxime A. Siegler, Huub Kooijman, Anthony L. Spek

**Affiliations:** a Bijvoet Center for Biomolecular Research, Crystal and Structural Chemistry, Utrecht University, Padualaan 8, 3584 CH Utrecht, The Netherlands

## Abstract

In the chiral title compound, C_18_H_28_O_3_, the two six-membered rings of the perhydronaphthalenone adopt a rigid chair–chair conformation and the five-membered dioxolanyl ring adopts an envelope conformation. The crystal structure is stabilized only by weak inter­actions.

## Related literature

For related literature, see Meulemans *et al.* (1999 ▶); Meulemans & de Groot (2007 ▶); Cremer & Pople (1975 ▶).
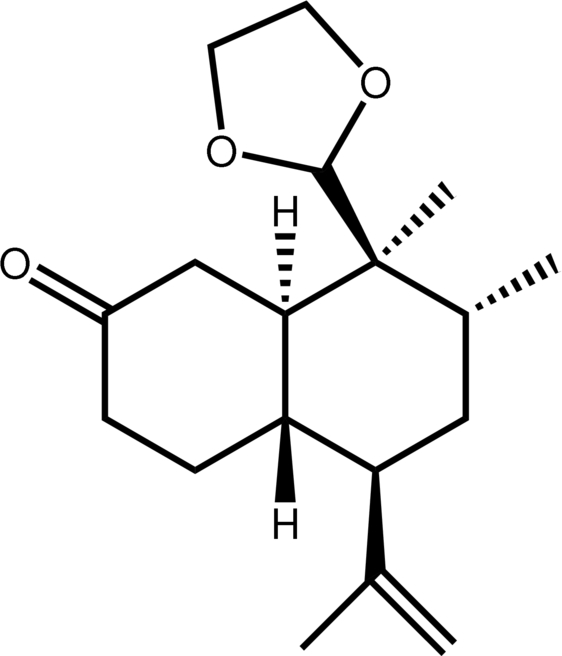


## Experimental

### 

#### Crystal data

C_18_H_28_O_3_
*M* *_r_* = 292.40
Monoclinic, 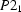

*a* = 8.9172 (9) Å
*b* = 11.0318 (12) Å
*c* = 8.9616 (9) Å
β = 116.354 (6)°
*V* = 789.95 (14) Å^3^
*Z* = 2
Mo *K*α radiation
μ = 0.08 mm^−1^
*T* = 150 (2) K
0.30 × 0.30 × 0.30 mm

#### Data collection

Nonius KappaCCD diffractometer
Absorption correction: none
6725 measured reflections
1678 independent reflections
1623 reflections with *I* > 2σ(*I*)
*R*_int_ = 0.034

#### Refinement

*R*[*F* ^2^ > 2σ(*F* ^2^)] = 0.028
*wR*(*F* ^2^) = 0.073
*S* = 1.07
1678 reflections
193 parameters
1 restraint
H-atom parameters constrained
Δρ_max_ = 0.21 e Å^−3^
Δρ_min_ = −0.12 e Å^−3^

### 

Data collection: *COLLECT* (Nonius, 1998 ▶); cell refinement: *DENZO* (Otwinowski & Minor, 1997 ▶); data reduction: *DENZO*; program(s) used to solve structure: *SHELXS86* (Sheldrick, 1985 ▶); program(s) used to refine structure: *SHELXL97* (Sheldrick, 1997 ▶); molecular graphics: *PLATON* (Spek, 2003 ▶); software used to prepare material for publication: *PLATON*.

## Supplementary Material

Crystal structure: contains datablocks I, global. DOI: 10.1107/S1600536807067335/si2067sup1.cif

Structure factors: contains datablocks I. DOI: 10.1107/S1600536807067335/si2067Isup2.hkl

Additional supplementary materials:  crystallographic information; 3D view; checkCIF report

## Figures and Tables

**Fig. 1.**
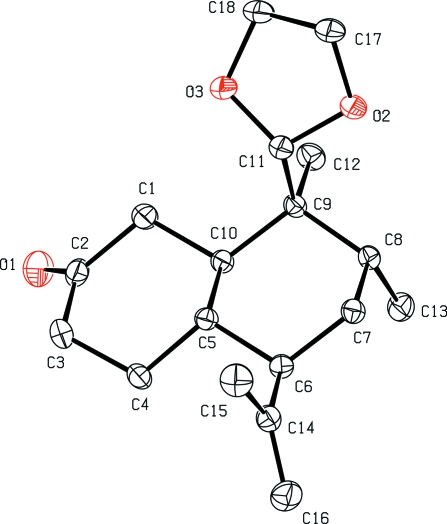
Displacement ellipsoid plot (50% probability level) of the asymmetric unit of the title compound at 150 K. H atoms are omitted for clarity.

**Fig. 2.**
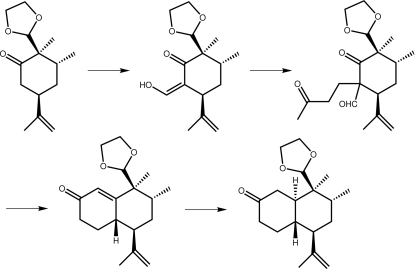
The formation of the title compound.

## supplementary crystallographic information

### Comment

Crystals of the title compound (Fig. 1) were obtained as an undesired product as part of an attempt to synthesize a clerodane diterpenoid. The stereochemistry at each of the chiral centers (*i.e.*, C5:*S*, C6:*R*, C8:*R*, C9:*S*, C10:*R*) was assigned based on the known chirality of C6:*R* of the starting material [*i.e.*, *R*-(-)-carvone] (Meulemans *et al.*,1999).

The ring (C1/C2—C10) adopts a chair conformation with puckering parameters of *Q* = 0.559 (2) Å, *θ* = 173.5 (2)°, *φ* = 78.4(1.7)° (Cremer & Pople, 1975). The ring (C5/C6—C10) adopts a chair conformation with puckering parameters *Q* = 0.559 (2) Å, *θ* =176.2 (2)°, *φ* = 359 (3)°. The two six-membered rings of the hydronaphtalenone derivative adopt a rigid chair-chair conformation (*i.e.*, the axial H atoms of the atoms C5 and C10 are located respectively below and above the best molecular plane of the octahydronaphtalen-2-one derivative).

The five-membered dioxolanyl ring (C11/O2—O3) adopts an envelope conformation on C17 (*i.e.*, the atoms C11, C18, O2 and O3 are coplanar and C17 projects out of the plane) with puckering parameters of *Q* = 0.340 (2)Å and *φ* = 152.0 (3)°. The torsion angles C17 – O2 – C11 – O3 and C18 – O3 – C11 – O2 are respectively 26.80 (16) and -5.37 (17). Weak interactions are found between the equatorial H atom of atom C1 and atom C18 [C1–H1B···C18 (1 - *x*, 1/2 + *y*, -*z*) = 2.88 Å].

Other short contacts [C5···O1 (2 - *x*, -1/2 + *y*, -*z*) = 3.680 (2) Å and C13···O1 (2 - *x*, -1/2 + *y*, 1 - *z*) = 3.503 (2) Å] are also found in the crystal structure.

### Experimental

Crystal of the title compound were obtained (Scheme 1) from Meulemans & de Groot (2007).

### Refinement

In the absence of significant anomalous scattering effects, Friedel pairs were merged prior to the refinement. The reflection 100 has been omitted from the refinement due to X-ray truncation at θ_min_ = 2.54° (θ_100_ = 2.55°). H atoms were found in difference Fourier maps and subsequently placed at calculated positions (C—H = 0.95–1.00 Å) with isotropic displacement parameters having values 1.2 or 1.5 times *U*_eq_ of the attached C atom.

### Crystal data

**Table:**

C_18_H_28_O_3_	*F*_000_ = 320
*M**_r_* = 292.40	*D*_x_ = 1.229 Mg m^−^^3^
Monoclinic, *P*2_1_	Mo *K*α radiation λ = 0.71073 Å
Hall symbol: P 2yb	Cell parameters from 225 reflections
*a* = 8.9172 (9) Å	θ = 2.0–20.0º
*b* = 11.0318 (12) Å	µ = 0.08 mm^−^^1^
*c* = 8.9616 (9) Å	*T* = 150 (2) K
β = 116.354 (6)º	Block, colourless
*V* = 789.95 (14) Å^3^	0.30 × 0.30 × 0.30 mm
*Z* = 2	

### Data collection

**Table:**

Nonius KappaCCD diffractometer	1623 reflections with *I* > 2σ(*I*)
Radiation source: rotating anode	*R*_int_ = 0.034
Monochromator: graphite	θ_max_ = 26.3º
*T* = 150(2) K	θ_min_ = 2.5º
φ and ω scans	*h* = −11→11
Absorption correction: none	*k* = −13→13
6725 measured reflections	*l* = −11→10
1678 independent reflections	

### Refinement

**Table:**

Refinement on *F*^2^	Secondary atom site location: difference Fourier map
Least-squares matrix: full	Hydrogen site location: difference Fourier map
*R*[*F*^2^ > 2σ(*F*^2^)] = 0.028	H-atom parameters constrained
*wR*(*F*^2^) = 0.074	*w* = 1/[σ^2^(*F*_o_^2^) + (0.0415*P*)^2^ + 0.0975*P*] where *P* = (*F*_o_^2^ + 2*F*_c_^2^)/3
*S* = 1.07	(Δ/σ)_max_ < 0.001
1678 reflections	Δρ_max_ = 0.21 e Å^−^^3^
193 parameters	Δρ_min_ = −0.12 e Å^−^^3^
1 restraint	Extinction correction: none
Primary atom site location: structure-invariant direct methods	Absolute structure: known chirality of atom C6(R)

### Special details

**Table:**

Geometry. All e.s.d.'s (except the e.s.d. in the dihedral angle between two l.s. planes) are estimated using the full covariance matrix. The cell e.s.d.'s are taken into account individually in the estimation of e.s.d.'s in distances, angles and torsion angles; correlations between e.s.d.'s in cell parameters are only used when they are defined by crystal symmetry. An approximate (isotropic) treatment of cell e.s.d.'s is used for estimating e.s.d.'s involving l.s. planes.
Refinement. Refinement of *F*^2^ against ALL reflections. The weighted *R*-factor *wR* and goodness of fit *S* are based on *F*^2^, conventional *R*-factors *R* are based on *F*, with *F* set to zero for negative *F*^2^. The threshold expression of *F*^2^ > 2σ(*F*^2^) is used only for calculating *R*-factors(gt) *etc*. and is not relevant to the choice of reflections for refinement. *R*-factors based on *F*^2^ are statistically about twice as large as those based on *F*, and *R*- factors based on ALL data will be even larger.

### Fractional atomic coordinates and isotropic or equivalent isotropic displacement parameters (Å^2^)

**Table:**

	*x*	*y*	*z*	*U*_iso_*/*U*_eq_	
O1	0.91230 (16)	1.20580 (12)	0.02689 (16)	0.0341 (3)	
O2	0.71825 (13)	0.66981 (11)	0.28172 (14)	0.0219 (3)	
O3	0.66971 (14)	0.78772 (12)	0.05496 (14)	0.0252 (3)	
C1	0.8606 (2)	1.01128 (16)	0.1128 (2)	0.0232 (4)	
H1A	0.8318	0.9447	0.0306	0.028*	
H1B	0.7548	1.0492	0.0998	0.028*	
C2	0.9642 (2)	1.10439 (16)	0.07722 (19)	0.0226 (4)	
C3	1.1366 (2)	1.06287 (16)	0.1097 (2)	0.0250 (4)	
H3A	1.2019	1.1327	0.1008	0.030*	
H3B	1.1282	1.0022	0.0250	0.030*	
C4	1.22676 (19)	1.00670 (15)	0.2844 (2)	0.0209 (3)	
H4A	1.3350	0.9725	0.2987	0.025*	
H4B	1.2510	1.0713	0.3687	0.025*	
C5	1.12471 (18)	0.90660 (14)	0.31573 (19)	0.0157 (3)	
H5A	1.1045	0.8400	0.2333	0.019*	
C6	1.22433 (18)	0.85501 (14)	0.49250 (19)	0.0166 (3)	
H6A	1.2545	0.9245	0.5723	0.020*	
C7	1.11779 (18)	0.76665 (14)	0.53733 (19)	0.0172 (3)	
H7A	1.1830	0.7396	0.6540	0.021*	
H7B	1.0928	0.6943	0.4649	0.021*	
C8	0.95238 (19)	0.82285 (14)	0.51790 (19)	0.0162 (3)	
H8A	0.8869	0.7565	0.5376	0.019*	
C9	0.84606 (18)	0.86925 (14)	0.33675 (19)	0.0160 (3)	
C10	0.95399 (18)	0.95839 (14)	0.29051 (19)	0.0159 (3)	
H10A	0.9796	1.0286	0.3686	0.019*	
C11	0.79211 (18)	0.75823 (15)	0.21959 (19)	0.0183 (3)	
H11A	0.8927	0.7225	0.2142	0.022*	
C12	0.68673 (19)	0.93240 (15)	0.3237 (2)	0.0218 (3)	
H12A	0.6360	0.8833	0.3804	0.033*	
H12B	0.6070	0.9416	0.2061	0.033*	
H12C	0.7157	1.0125	0.3762	0.033*	
C13	0.9859 (2)	0.91914 (16)	0.6532 (2)	0.0229 (3)	
H13A	1.0418	0.9891	0.6327	0.034*	
H13B	1.0576	0.8846	0.7627	0.034*	
H13C	0.8795	0.9450	0.6503	0.034*	
C14	1.38701 (19)	0.79469 (15)	0.51502 (19)	0.0196 (3)	
C15	1.3778 (2)	0.70016 (18)	0.3920 (2)	0.0281 (4)	
H15A	1.3457	0.7380	0.2831	0.042*	
H15B	1.2942	0.6391	0.3826	0.042*	
H15C	1.4874	0.6613	0.4294	0.042*	
C16	1.5329 (2)	0.82505 (18)	0.6422 (2)	0.0284 (4)	
H16A	1.6332	0.7863	0.6555	0.034*	
H16B	1.5362	0.8854	0.7192	0.034*	
C17	0.5970 (2)	0.60851 (16)	0.1386 (2)	0.0257 (4)	
H17A	0.5079	0.5721	0.1616	0.031*	
H17B	0.6495	0.5443	0.1004	0.031*	
C18	0.5291 (2)	0.70922 (18)	0.0126 (2)	0.0290 (4)	
H18A	0.4889	0.6777	−0.1022	0.035*	
H18B	0.4360	0.7519	0.0221	0.035*	

### Atomic displacement parameters (Å^2^)

**Table:**

	*U*^11^	*U*^22^	*U*^33^	*U*^12^	*U*^13^	*U*^23^
O1	0.0380 (7)	0.0257 (7)	0.0346 (7)	0.0021 (6)	0.0126 (6)	0.0121 (6)
O2	0.0228 (6)	0.0192 (6)	0.0210 (6)	−0.0074 (5)	0.0073 (5)	−0.0009 (5)
O3	0.0228 (6)	0.0278 (6)	0.0189 (6)	−0.0096 (5)	0.0037 (5)	0.0007 (5)
C1	0.0176 (7)	0.0256 (8)	0.0229 (8)	−0.0005 (7)	0.0059 (6)	0.0072 (7)
C2	0.0269 (8)	0.0228 (8)	0.0156 (7)	−0.0027 (7)	0.0072 (6)	0.0028 (7)
C3	0.0265 (8)	0.0273 (9)	0.0235 (8)	−0.0047 (7)	0.0132 (7)	0.0047 (7)
C4	0.0177 (7)	0.0220 (8)	0.0237 (8)	−0.0019 (6)	0.0100 (6)	0.0043 (7)
C5	0.0149 (7)	0.0154 (7)	0.0163 (7)	−0.0006 (6)	0.0064 (6)	0.0000 (6)
C6	0.0162 (7)	0.0167 (7)	0.0170 (7)	0.0007 (6)	0.0074 (6)	0.0003 (6)
C7	0.0175 (7)	0.0149 (7)	0.0186 (7)	0.0008 (6)	0.0073 (6)	0.0024 (6)
C8	0.0172 (7)	0.0153 (7)	0.0167 (7)	−0.0011 (6)	0.0081 (6)	0.0011 (6)
C9	0.0147 (7)	0.0165 (7)	0.0179 (7)	0.0000 (6)	0.0081 (6)	0.0012 (6)
C10	0.0146 (7)	0.0149 (7)	0.0172 (7)	0.0008 (6)	0.0063 (6)	0.0027 (6)
C11	0.0175 (7)	0.0183 (7)	0.0181 (7)	−0.0020 (6)	0.0071 (6)	0.0000 (6)
C12	0.0177 (7)	0.0226 (8)	0.0266 (8)	0.0027 (6)	0.0111 (6)	0.0018 (7)
C13	0.0258 (8)	0.0242 (8)	0.0205 (8)	−0.0013 (7)	0.0120 (7)	−0.0027 (7)
C14	0.0182 (7)	0.0197 (8)	0.0215 (8)	0.0028 (6)	0.0093 (6)	0.0042 (6)
C15	0.0262 (8)	0.0277 (9)	0.0330 (9)	0.0070 (7)	0.0155 (7)	0.0005 (8)
C16	0.0204 (8)	0.0364 (10)	0.0267 (9)	0.0069 (7)	0.0088 (7)	0.0043 (8)
C17	0.0239 (8)	0.0245 (8)	0.0242 (8)	−0.0097 (7)	0.0067 (7)	−0.0038 (7)
C18	0.0205 (8)	0.0330 (9)	0.0262 (8)	−0.0104 (7)	0.0039 (7)	0.0012 (8)

### Geometric parameters (Å, °)

**Table:**

O1—C2	1.218 (2)	C8—C9	1.559 (2)
O2—C11	1.4215 (19)	C8—H8A	1.0000
O2—C17	1.428 (2)	C9—C12	1.539 (2)
O3—C18	1.430 (2)	C9—C11	1.544 (2)
O3—C11	1.4298 (18)	C9—C10	1.5554 (19)
C1—C2	1.507 (2)	C10—H10A	1.0000
C1—C10	1.547 (2)	C11—H11A	1.0000
C1—H1A	0.9900	C12—H12A	0.9800
C1—H1B	0.9900	C12—H12B	0.9800
C2—C3	1.503 (2)	C12—H12C	0.9800
C3—C4	1.537 (2)	C13—H13A	0.9800
C3—H3A	0.9900	C13—H13B	0.9800
C3—H3B	0.9900	C13—H13C	0.9800
C4—C5	1.534 (2)	C14—C16	1.337 (2)
C4—H4A	0.9900	C14—C15	1.493 (2)
C4—H4B	0.9900	C15—H15A	0.9800
C5—C6	1.541 (2)	C15—H15B	0.9800
C5—C10	1.5458 (19)	C15—H15C	0.9800
C5—H5A	1.0000	C16—H16A	0.9500
C6—C14	1.526 (2)	C16—H16B	0.9500
C6—C7	1.534 (2)	C17—C18	1.506 (3)
C6—H6A	1.0000	C17—H17A	0.9900
C7—C8	1.537 (2)	C17—H17B	0.9900
C7—H7A	0.9900	C18—H18A	0.9900
C7—H7B	0.9900	C18—H18B	0.9900
C8—C13	1.538 (2)		
			
C11—O2—C17	105.72 (12)	C11—C9—C8	108.03 (12)
C18—O3—C11	108.30 (12)	C10—C9—C8	108.84 (11)
C2—C1—C10	111.98 (13)	C5—C10—C1	109.58 (12)
C2—C1—H1A	109.2	C5—C10—C9	114.40 (12)
C10—C1—H1A	109.2	C1—C10—C9	113.50 (12)
C2—C1—H1B	109.2	C5—C10—H10A	106.2
C10—C1—H1B	109.2	C1—C10—H10A	106.2
H1A—C1—H1B	107.9	C9—C10—H10A	106.2
O1—C2—C3	122.63 (16)	O2—C11—O3	106.63 (12)
O1—C2—C1	122.44 (16)	O2—C11—C9	109.67 (12)
C3—C2—C1	114.93 (14)	O3—C11—C9	112.69 (13)
C2—C3—C4	110.36 (13)	O2—C11—H11A	109.3
C2—C3—H3A	109.6	O3—C11—H11A	109.3
C4—C3—H3A	109.6	C9—C11—H11A	109.3
C2—C3—H3B	109.6	C9—C12—H12A	109.5
C4—C3—H3B	109.6	C9—C12—H12B	109.5
H3A—C3—H3B	108.1	H12A—C12—H12B	109.5
C5—C4—C3	113.07 (12)	C9—C12—H12C	109.5
C5—C4—H4A	109.0	H12A—C12—H12C	109.5
C3—C4—H4A	109.0	H12B—C12—H12C	109.5
C5—C4—H4B	109.0	C8—C13—H13A	109.5
C3—C4—H4B	109.0	C8—C13—H13B	109.5
H4A—C4—H4B	107.8	H13A—C13—H13B	109.5
C4—C5—C6	109.63 (12)	C8—C13—H13C	109.5
C4—C5—C10	109.45 (12)	H13A—C13—H13C	109.5
C6—C5—C10	111.58 (11)	H13B—C13—H13C	109.5
C4—C5—H5A	108.7	C16—C14—C15	121.22 (15)
C6—C5—H5A	108.7	C16—C14—C6	120.78 (15)
C10—C5—H5A	108.7	C15—C14—C6	118.00 (14)
C14—C6—C7	110.56 (12)	C14—C15—H15A	109.5
C14—C6—C5	112.18 (12)	C14—C15—H15B	109.5
C7—C6—C5	111.26 (12)	H15A—C15—H15B	109.5
C14—C6—H6A	107.5	C14—C15—H15C	109.5
C7—C6—H6A	107.5	H15A—C15—H15C	109.5
C5—C6—H6A	107.5	H15B—C15—H15C	109.5
C6—C7—C8	112.88 (12)	C14—C16—H16A	120.0
C6—C7—H7A	109.0	C14—C16—H16B	120.0
C8—C7—H7A	109.0	H16A—C16—H16B	120.0
C6—C7—H7B	109.0	O2—C17—C18	102.48 (14)
C8—C7—H7B	109.0	O2—C17—H17A	111.3
H7A—C7—H7B	107.8	C18—C17—H17A	111.3
C7—C8—C13	110.40 (12)	O2—C17—H17B	111.3
C7—C8—C9	111.09 (12)	C18—C17—H17B	111.3
C13—C8—C9	114.17 (13)	H17A—C17—H17B	109.2
C7—C8—H8A	106.9	O3—C18—C17	103.57 (13)
C13—C8—H8A	106.9	O3—C18—H18A	111.0
C9—C8—H8A	106.9	C17—C18—H18A	111.0
C12—C9—C11	107.98 (12)	O3—C18—H18B	111.0
C12—C9—C10	110.66 (12)	C17—C18—H18B	111.0
C11—C9—C10	111.29 (12)	H18A—C18—H18B	109.0
C12—C9—C8	110.00 (12)		
			
C10—C1—C2—O1	−126.84 (17)	C2—C1—C10—C5	−55.17 (18)
C10—C1—C2—C3	52.74 (19)	C2—C1—C10—C9	175.58 (13)
O1—C2—C3—C4	129.35 (17)	C12—C9—C10—C5	174.62 (12)
C1—C2—C3—C4	−50.2 (2)	C11—C9—C10—C5	−65.32 (16)
C2—C3—C4—C5	52.91 (19)	C8—C9—C10—C5	53.62 (16)
C3—C4—C5—C6	179.67 (13)	C12—C9—C10—C1	−58.63 (17)
C3—C4—C5—C10	−57.64 (17)	C11—C9—C10—C1	61.44 (16)
C4—C5—C6—C14	−63.06 (16)	C8—C9—C10—C1	−179.62 (12)
C10—C5—C6—C14	175.53 (13)	C17—O2—C11—O3	26.80 (16)
C4—C5—C6—C7	172.49 (12)	C17—O2—C11—C9	149.10 (12)
C10—C5—C6—C7	51.08 (16)	C18—O3—C11—O2	−5.37 (17)
C14—C6—C7—C8	179.67 (12)	C18—O3—C11—C9	−125.73 (14)
C5—C6—C7—C8	−54.97 (17)	C12—C9—C11—O2	−67.98 (15)
C6—C7—C8—C13	−70.08 (16)	C10—C9—C11—O2	170.38 (12)
C6—C7—C8—C9	57.63 (16)	C8—C9—C11—O2	50.96 (15)
C7—C8—C9—C12	−176.34 (13)	C12—C9—C11—O3	50.63 (16)
C13—C8—C9—C12	−50.71 (16)	C10—C9—C11—O3	−71.01 (15)
C7—C8—C9—C11	66.02 (15)	C8—C9—C11—O3	169.56 (11)
C13—C8—C9—C11	−168.35 (12)	C7—C6—C14—C16	−107.18 (17)
C7—C8—C9—C10	−54.94 (15)	C5—C6—C14—C16	127.98 (17)
C13—C8—C9—C10	70.69 (15)	C7—C6—C14—C15	72.20 (18)
C4—C5—C10—C1	57.34 (16)	C5—C6—C14—C15	−52.63 (19)
C6—C5—C10—C1	178.86 (13)	C11—O2—C17—C18	−36.40 (16)
C4—C5—C10—C9	−173.90 (12)	C11—O3—C18—C17	−16.77 (18)
C6—C5—C10—C9	−52.38 (16)	O2—C17—C18—O3	32.42 (17)
